# Diversity and pathogenesis of *Staphylococcus aureus* from bovine mastitis: current understanding and future perspectives

**DOI:** 10.1186/s12917-022-03197-5

**Published:** 2022-03-24

**Authors:** Bruno Campos, Amy C. Pickering, Lis Souza Rocha, Ananda Pereira Aguilar, Mary Hellen Fabres-Klein, Tiago Antônio de Oliveira Mendes, J. Ross Fitzgerald, Andrea de Oliveira Barros Ribon

**Affiliations:** 1 Departamento de Bioquímica e Biologia Molecular, Universidade Federal de Viçosa, Minas Gerais Viçosa, Brazil; 2 The Roslin Institute, Royal (Dick) School of Veterinary Studies, University of Edinburgh, Easter Bush Campus, Edinburgh, UK; 3 Centro de Ciências Biológicas e da Saúde, Universidade Federal do Oeste da Bahia, Barreiras, Bahia Brazil

**Keywords:** *Staphylococcus aureus*, Bovine mastitis, Clonal complexes, Pathogenesis

## Abstract

*Staphylococcus aureus* is a leading cause of bovine mastitis worldwide. Despite some improved understanding of disease pathogenesis, progress towards new methods for the control of intramammary infections (IMI) has been limited, particularly in the field of vaccination. Although herd management programs have helped to reduce the number of clinical cases, *S. aureus* mastitis remains a major disease burden. This review summarizes the past 16 years of research on bovine *S. aureus* population genetics, and molecular pathogenesis that have been conducted worldwide. We describe the diversity of *S. aureus* associated with bovine mastitis and the geographical distribution of *S. aureus* clones in different continents. We also describe studies investigating the evolution of bovine *S. aureus* and the importance of host-adaptation in its emergence as a mastitis pathogen. The available information on the prevalence of virulence determinants and their functional relevance during the pathogenesis of bovine mastitis are also discussed. Although traits such as biofilm formation and innate immune evasion are critical for the persistence of bacteria, the current understanding of the key host-pathogen interactions that determine the outcome of *S. aureus* IMI is very limited. We suggest that greater investment in research into the genetic and molecular basis of bovine *S. aureus* pathogenesis is essential for the identification of novel therapeutic and vaccine targets.

## Background

Bovine mastitis is a multifactorial inflammatory disease that depends on a combination of animal-, environmental-, and pathogen-related factors. Visible abnormalities in the milk, swelling or tenderness of the udder are signs of clinical mastitis while no overt signs are observed in subclinical mastitis. *S. aureus* is a well-studied opportunistic pathogen frequently associated with subclinical mastitis and responsible for large economic losses due to reduced milk quality and production [1]. *S. aureus* spreads among cows during milking, requiring a cooperative approach to reduce dissemination to healthy animals [2]. Bovine isolates of *S. aureus* are also a leading cause of foodborne diseases with bulk tank and raw milk products important vehicles for bacterial transmission to humans [3].

The importance of the human-animal-environment One Health approach for investigating disease transmission and control has been well highlighted [4] but concrete strategies are still needed to reduce the burden of infectious diseases and the impact of antimicrobial resistance in livestock and humans. Indeed, implementation of the One Health approach is essential to improve animal welfare, enhance food safety, and promote human health. An important consideration is the capacity for transmission of *S. aureus* among livestock and humans or host-switching events leading to the emergence of new pathogenic or resistant clones [5, 6].

**Fig. 1:**
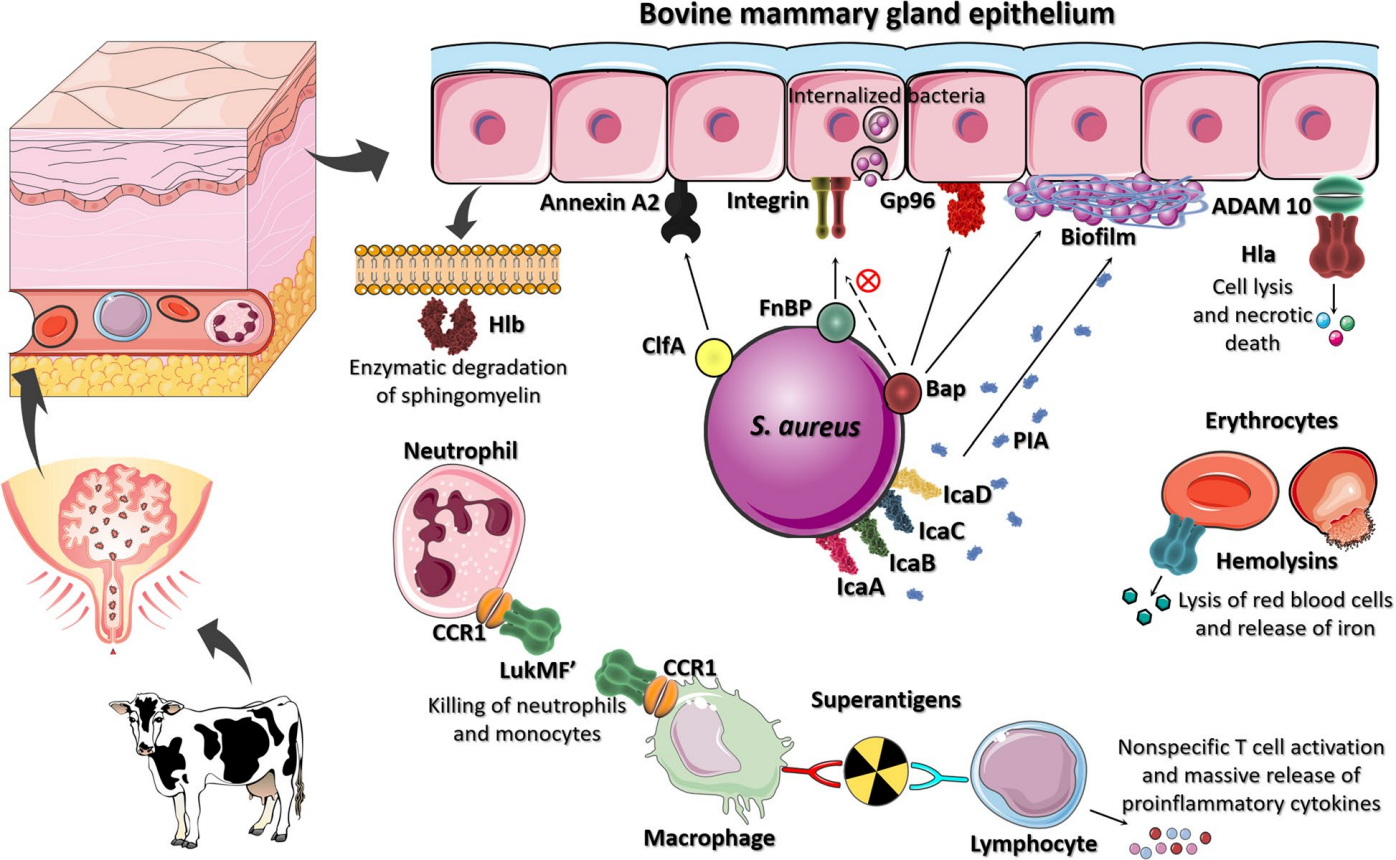
Proteins that have been implicated in the pathogenesis of *Staphylococcus aureus* of bovine origin. Surface proteins interact with host proteins to promote bacterial adhesion and invasion. Biofilm-associated protein (Bap) interferes in the internalization pathway mediated by FnBP and contributes to biofilm formation, a process that also depends on the *ica* locus. Toxins such as alpha and beta hemolysins, promote necrosis of the mammary gland tissue and lyse bovine erythrocytes to use hemoglobin as a source of iron. Leukotoxin LukMF´ binds to neutrophils present in the bovine milk. Staphylococcal superantigens activate T-cells resulting in the release of various pro-inflammatory cytokines. Figure created by authors using resources from Smart Servier Medical Art and BioRender

*S. aureus* of bovine origin has long been a focus of microbiological research. However, the scientific knowledge gained has yet to be translated into effective vaccines, therapeutics or rapid, inexpensive diagnostics that can be applied for better disease control [7]. Limited insights into the pathogenesis of mastitis will result from analysis of *S. aureus* strains in isolation. It is imperative that we examine the bacterial interaction with the host to identify novel therapeutics to fight this old disease. This review has compiled worldwide studies on the population diversity and virulence of *S. aureus* of bovine origin that have been published in the last 16 years. Articles describing the clonal complexes (CCs) or *spa* types identified among bovine *S. aureus* were included to reveal the worldwide distribution of the pathogen. Overall, there are remarkable differences in virulence profiles among field isolates and some virulence factors display ruminant-specific features like LukMF’ suggesting a potential role during intramammary infection (IMI). Studies that shed light on the mechanism of virulence factors made by *S. aureus* from bovine IMI are described with emphasis on adhesins and toxins whose function has been experimentally demonstrated (Fig. 1).

### Main text

#### Worldwide distribution of clones

From 2004 to early 2021, 79 articles were published on the epidemiology of bovine *S. aureus* isolated in 31 countries. However, the 67 articles that used multilocus sequence typing (MLST) or *spa* typing, two commonly used methods for the molecular typing of *S. aureus* isolates, were included in this review (Table 1). CC97 was the most disseminated genotype, identified in 15 different countries of America, Asia, Europe, and Africa. CC97 has been reported as the dominant lineage in Chile [8], Brazil [9, 10], Japan [11], and the USA [8]. It has been reported that IMI cases caused by CC97 strains lead to asymptomatic, subclinical or persistent infections, increasing the challenge of pathogen control in dairy herds [11, 12]. Based on genomic comparison, a higher virulence potential was predicted for CCs 151 and 97 compared to other bovine-associated *S. aureus* lineages [13], a finding that is consistent with functional and *in vivo* studies carried out [14, 15].

**Table 1:** Clonal complexes and spa types of bovine isolates of *Staphylococcus aureus* disseminated worldwide

Countries	Clonal complex	spa types	References
**Algeria**	CC8, CC97	t024, t267, t359, t1965, t521, t2112, t7234, t11511	[16, 17]
**Austria**	ND	t011, t024, t095, t097, t359, t398, t521, t524, t529, t1182, t1403, t2953, t6158, t13487, t16197, t16200	[18]
**Belgium**	CC8, CC398	t008, t011, t037, t108, t121, t388, t567, t1451, t1456, t1985, t3423, t6228	[19, 20]
**Brazil**	CC1, CC5, CC30, CC97, CC126, CC133	t002, t021, t114, t127, t138, t177, t267, t318, t321, t342, t359, t456, t458, t521, t559, t605, t693, t1192, t2066, t2164, t3324, t6811, t6980, t7335, t10856, t11659	[9, 10, 21, 22]
**Canada**	CC8, CC97, CC126, CC133, CC151	t015, t021, t127, t177, t224, t267, t359, t451, t521, t529, t605, t1166, t1190, t1236, t1965, t2211, t2445, t3051, t3380, t10610, t11215, t12186, t13401, t16275	[13, 23, 24]
**Chile**	CC97	ND	[8]
**China**	CC1, CC7, CC50, CC81, CC88, CC97, CC188, CC398, CC479, CC705, CC5405, CC5406	t034, t127, t131, t189, t224, t237, t267, t359, t518, t519, t521, t528, t529, t571, t730, t1234, t1764, t2279, t2592, t2699, t2756, t2970, t4570, t4682, t5100, t6297, t14156, t16314, t16315, t17095, t17182	[25–27]
**Egypt**	CC5, CC15, CC88, CC188, CC398	t084, t127, t167, t223, t267, t304, t314, t359 t786, t1234, t2117, t3071, t4019	[28, 29]
**Finland**	ND	t172, t3256	[30]
**Germany**	CC1, CC5, CC8, CC20, CC50, CC97, CC130, CC133, CC151, CC350, CC398, CC479, CC705	t011, t034, t91, t267, t359, t519, t521, t524, t528, t529, t571, t586, t1106, t1403, t2576, t2873, t3297, t5180, t5920, t10610, t13769	[31–38]
**Hungary**	CC1	t127	[39]
**India**	ND	t002, t008, t267, t304, t311, t359, t1200, t2104, t2478, t2770, t2802, t2915, t3841 t3992, t5019, t6297, t6861, t6877, t7286, t7287, t7288, t7680, t7681, t7683, t7684, t7695, t7696, t7867	[40–42]
**Iran**	CC5, CC22, CC45, CC88, CC398, CC522,	t084, t230, t267, t304, t521, t527, t937, t2526, t3576, t3680	[43]
**Ireland**	CC97, CC151, CC126	ND	[44]
**Israel**	CC1, CC5, CC7, CC12, CC15, CC20, CC88, CC151, CC188	t127, t002, t091, t160, t164, t186, t529, t189, t3380	[31]
**Italy**	CC1, CC5, CC8, CC20, CC72, CC97, CC101, CC126, CC133, CC151, CC398, CC479, CC522, CC705	t011, t024, t034, t053, t108, t127, t164, t267, t309, t359, t442, t521, t524, t529, t535, t548, t605, t688, t730, t899, t1236, t2421, t2953, t3802, t3987, t4795, t5268, t5694, t9295, t13277, t13269, t13278	[31, 45–50]
**Japan**	CC5, CC6, CC7, CC8, CC12, CC15, CC20, CC25,CC30,CC45,CC59, CC88, CC97, CC509, CC705	t002, t008, t021, t024, t050, t078, t091, t127, t160, t164, t179, t189, t203, t216, t224, t258, t267, t287, t359, t362, t375, t377, t458, t521, t529, t630, t693, t701, t729, t881, t1028, t1109, t1201, t1234, t1767, t1775, t1858, t2109, t2360, t2453, t2844, t3277, t3332, t3418, t3782, t3929, t4133, t4359, t4542, t5259, t5260, t5261, t5263, t5264, t5265, t5266, t5267, t5352, t5412	[11, 12, 51, 52]
**Korea**	ND	t002, t034, t084, t127, t148, t164, t189, t304, t324, t2459, t2612, t4050	[53–55]
**Norway**	CC1, CC2, CC3, CC5, CC25, CC133, CC135, CC136, CC479	ND	[56]
**Poland**	CC97	ND	[57]
**Portugal**	CC7, CC97, CC133	t091, t267, t1166	[58]
**Rwanda**	CC97, CC152, CC3591, CC3666	t355, t458, t1236, t1398, t2112, t9432, t10103, t18835, t18853	[59]
**Sweden**	ND	t008, t052, t127, t164, t177, t267, t359, t529, t543, t1294, t1403, t4955, t13621	[60]
**Switzerland**	CC1, CC5, CC7, CC8, CC9, CC15, CC20, CC45, CC97, CC151, CC398, CC479, CC705	t018, t024, t034, t052, t160, t164, t267, t458, t524, t529, t543, t711, t2094, t2953, t3802, t4318, t5268, t5270, t5271, t5694, t6281, t7007, t7013, t7061, t12926	[37, 61–70]
**Tunisia**	CC1, CC6, CC15, CC97, CC188, CC522, CC1153	t267, t426, t509, t521, t529, t1773, t2421, t2802, t2844, t10381	[71, 72]
**Uganda**	CC435	t645	[73]
**UK**	CC9, CC97, CC130, CC151, CC425	t131, t224, t267, t359, t521, t529, t742, t843, t6220, t6292, t6300, t8433	[8, 74, 75]
**USA**	CC1, CC5, CC8, CC9, CC25, CC30, CC45, CC50, CC51, CC97, CC126, CC188, CC206, CC258, CC348, CC350, CC353, CC354, CC398, CC705	t034, t044, t084, t189, t203, t267, t337, t359, t521, t529, t1166, t2734, t4173	[8, 31, 76]

*ND* not determined

CC1 and CC5 also have a global distribution, each being reported among cows in ten countries. Although these lineages are associated with human infections [77, 78] they have recently emerged in cows via host switch events [79]. It is reported that CC1 is less likely to cause clinical mastitis in cows [80]. The CC126, CC130, and CC133 strains were less widely distributed, in four, two, and six countries, respectively. CC133 and CC130 were localized in Europe with CC130 found in Germany and the UK and CC133 identified in four countries (Portugal, Italy, Germany, and Norway) in addition to Brazil and Canada. Although CC133 is most related to small ruminants [31], it was also isolated from cows with mastitis [44, 81]. CC126 was identified in Brazil [21], the USA [8], and Italy [45] and frequently recovered from subclinical mastitis.

Several CCs such as CC97, CC705, CC398, CC479, and CC8 were shared between proximal countries Germany, Italy, and Switzerland, consistent with cross-border movement of animals. Of note, CC479 strains were associated with severe bovine mastitis cases [80, 82] and demonstrated to induce a stronger pro-inflammatory response from bovine mammary epithelial cells (bMEC) than other bovine associated lineages such as CC151 [83]. Unlike CC479 and CC705 that show high prevalence in only Northern Italy, Germany, and Switzerland [32, 33, 46, 47, 61, 62], methicillin-resistant isolates of CC398 have been reported in eight countries across three continents: the USA, China, Belgium, Egypt, Germany, Iran, Italy, and Switzerland [34, 43, 47, 48, 84]. The ST398 MRSA lineage emerged in pigs but is now widely identified in other animal hosts including ruminants, poultry, horses, dogs, and cats [85]. It is reported that CC398 is a more promiscuous clone compared to other CCs [86], and therefore may represent a greater threat to public and animal health, especially considering the prevalence of MRSA strains.

CC8 is a global human lineage associated with an array of different diseases and includes the major epidemic clone USA300 [87]. Recently, CC8 *S. aureus* strains have been found in association with bovines [88] and reported in eleven countries: Algeria, Australia, Belgium, Canada, China, Germany, Italy, Japan, Switzerland, and the USA. It was suggested that the bovine sequence type (ST) 8 strains were the likely result of a recent human to bovine host jump [63, 89]. These data are important to evaluate the transmission risk for people working in the farm and milking environments and for consumers of raw dairy products [35]. Studies conducted in Uganda and Hungary identified the same lineage in both milkers and cows, suggesting transmission events between species, although the direction of transfer (cow-human or human-cow) was not defined [39, 73].

Taken together, this literature review identified a wide variety of *S. aureus* genotypes in dairy cattle worldwide. Considering the region of interest, it might help to formulate strategies to understand and reduce the infection spread of *S. aureus* strains. The most disseminated genotypes (CCs) globally were CC97 (15 countries), CC1 (10 countries), CC5 (10 countries), CC8 (8 countries), and CC398 (8 countries). Some lineages may be the result of human to bovine host switching events.

### Population genomics of bovine *S. aureus* host-adaptation

The wide availability of inexpensive high-throughput sequencing has resulted in an explosion of whole genomes sequences for *S. aureus* and several population genomic studies of bovine strains have been published since the first genome of a bovine *S. aureus* strain RF122 was reported [90]. Overall, as indicated from population studies summarized in the previous section, a limited number of widely distributed *S. aureus* clones are responsible for the great majority of *S. aureus* mastitis cases worldwide [5, 86]. These clones have evolved through human to bovine host-switching events that have happened since the Neolithic era after domestication led to increased opportunities for transmission among humans and livestock [86, 91]. After a host-jump into cows from humans, *S. aureus* has undergone host-adaptation via gene acquisition, loss and diversification to allow it to survive in an anatomically and physiologically distinct niche [86, 90, 92]. For example, mobile genetic elements (MGE) including staphylococcal pathogenicity islands and bacteriophages which encode bovine-specific effectors of virulence are widely disseminated among bovine *S. aureus* clones and encode known and putative virulence factors including superantigens, von Willebrand binding protein (vWBP), and LukMF’ [92–94]. These effectors represent some of the best characterized factors involved in the pathogenesis of *S. aureus* mastitis with particular importance in both innate and acquired immune evasion [95, 96].

In addition to gene acquisition, loss of gene function has been a hallmark of bovine and ovine *S. aureus* strains that are adapting to their ruminant host species, as genes which are unessential for survival or fitness in the new host acquire nonsense or frameshift mutations that corrupt the open reading frame resulting in truncated or untranslated proteins [90, 92]. In addition, diversification of existing genes can occur that is associated with adaptation to a distinct host environment. For example, in bovine *S. aureus* strains, genes associated with carbohydrate utilization were identified to be under diversifying selective pressure suggesting adaptive evolution. Consistent with this genetic signature, bovine strains were better able to utilize lactose, the major source of carbohydrate in bovine milk, than *S. aureus* strains from humans or birds [86]. In another study, extensive recombination events were associated with the evolution of a subtype of CC97 (ST71), which led to the acquisition of several genes encoding proteins that promoted human innate immune evasion [97].

A recent study from Richardson et al. estimated the number of host-switching events that have occurred during the evolutionary history of *S. aureus* and although most jumps have occurred from humans into cows [89], several instances of reverse host-switches from cows back into humans have been identified [86]. For example, a study of the CC97 clone has identified at least two host-switch events from cows into humans that led to the emergence of a new human pathogenic clone that has spread around the world [6]. In addition, the major human epidemic clone in Southeast Asia (ST59) is predicted to have originated in cows [91, 98]. In each case, the strains have adapted to a human host species by the acquisition of genes required for human adaptation such as the immune evasion cluster of the Sa3int phage family which encodes factors that mediate human-specific innate immune evasion [6]. Richardson et al. identified a set of putative MGE that was associated with host-switching events suggesting an important role in host-adaptation [86] and providing avenues for future research into the host-pathogen interactions important for the colonization of dairy cows and the pathogenesis of mastitis.

### Pathogenesis of bovine *S. aureus*

#### Adhesion to bovine mammary cells

Colonization is a key step in bacterial pathogenesis and bovine *S. aureus* has evolved in many ways to facilitate adhesion to different host cell types [98, 99]. One family of *S. aureus* adhesins comprises the microbial surface component recognizing adhesive matrix molecules (MSCRAMMs), which are cell-wall anchored proteins that share structural features like an N-terminal folded domain responsible for ligand binding, and a wall-spanning region followed by a sorting signal located at the C-terminal that anchor the protein to the cell wall [100].

Adhesion to and invasion of bovine mammary epithelial cells is mainly promoted by the fibronectin-binding protein MSCRAMMs FnBPA and FnBPB, [101]. Fibronectin acts as a bridge that connects FnBPA to the α_5_β_1_ integrin present on the cell surface [102, 103]. It has been demonstrated that the FnBP-integrin interaction induces the assembly of a cytosolic protein complex that modulates cytoskeleton rearrangement and mediates bacterial uptake [104]. *S aureus* may also adhere to fibrinogen, elastin, and plasminogen in an FnBP-dependent manner [105–107] that could promote colonization of different tissues and spread to other anatomical sites. Laboratory strains lacking FnBPs have a lower ability to colonize mouse mammary glands under suckling pressure, confirming that FnBPs confer a competitive advantage *in vivo* and may be considered as virulence factors for mammary gland colonization [108]. Overexpression of the *fnb* genes results in a higher invasion of bovine mammary epithelial cells (bMEC) [109]. Expression of FnBPB but not FnBPA is also related to increased invasiveness of isolates representative of ST8 [110]. However, the presence of *fnb* genes is not a prerequisite for cell adhesion because bacteria still retain the ability to invade even if both genes are absent [99], or if a truncated protein is produced [110] suggesting that additional factors may also be involved.

The high prevalence reported for the *fnbA* gene in bovine isolates [111–113] may reflect the importance of this adhesin in the pathogenesis of bovine mastitis. In contrast, a considerable discrepancy (1.5–100%) in the prevalence of the *fnbB* gene was reported in bovine *S. aureus* isolates, even among isolates from the same region that may in part reflect allelic variation that precluded PCR amplification [25, 114, 115].

Bovine isolates of *S. aureu*s produce other MSCRAMMs that may promote colonization [109, 116, 117]. For example, clumping factors A and B (ClfA and ClfB) are fibrinogen-binding proteins that act as adhesins and have several defined roles in colonization and pathogenesis [100, 118, 119]. Although adherence to human endothelial cells requires fibrinogen to mediate the interaction between ClfA and the host integrin α5β3 [120], adherence to bovine epithelial cells occurs in a fibrinogen-independent manner via the annexin A2 receptor [121]. ClfA expressed by *S. aureus* also inhibits phagocytosis through the cleavage of C3b in a process mediated by an interaction with the complement regulator factor I [122]. It is yet to be confirmed if a similar inhibition mechanism is utilized by bovine *S. aureus* isolates. The *clfA* gene is usually described as highly prevalent (63.7-100%) in bacterial isolates of dairy cattle across all investigated countries with some disparities seen in the Netherlands (21%) [99, 123, 124]. The frequency of the *clfB* gene is reported as between 50 and 100% in bovine *S. aureus* isolates [25, 99, 111].

The collagen adhesin (Cna) protein is a MSCRAMM that not only has a role in adhesion but also participates in immune evasion during human infection [125]. Again, there is considerable variation in the prevalence of the *cna* gene observed among *S. aureus* isolates in dairy herds around the world [26, 111, 114]. Considering that collagen is highly prevalent in udder tissue, the expression of the *cna* gene by *S. aureus* of bovine origin could be important for adherence. Of note, it has been demonstrated that the *cna* gene has been acquired by the ST71 lineage of CC97 through recombination, conferring the capacity for adherence to collagen *in vitro* [97].

Other less studied fibrinogen-binding proteins have been described in *S. aureus* of bovine origin like the serine-aspartate repeat proteins (Sdr), extracellular fibrinogen binding protein (Efb), and the iron-regulated adhesin IsdA. Although there is no clear role for *sdrCDE* in mastitis the *sdrD* gene correlates with a high prevalence of IMI [126]. In addition, a strong association between the *srdD* and *sdrE* genes and clinical mastitis has been reported [127]. The *efb* gene has also been reported to have a high prevalence in field isolates [123]. During human infection, the interaction of Efb with fibrinogen creates a protective shield with an anti-phagocytic role indicating an immunosuppressive role [53]. Although IsdA binds to fibrinogen and fibronectin [128], it has been well-studied as a vaccine target for mastitis control along with other iron-regulated proteins such as IsdB and IsdH due to their high immunogenicity, gene expression during IMI and presence of antibodies in milk of animals naturally infected with *S. aureus* [7, 129, 130].

Bovine isolates of *S. aureus* produce a carbohydrate-based surface component when cultured in milk whey resulting in enhancement of bacterial adherence to bovine mammary cells and increased virulence in a murine model of mastitis [131, 132]. Although the carbohydrate-based surface component was never confirmed as a true capsule, other authors described encapsulated isolates as less adherent compared to acapsular strains [133, 134]. Internalization by MAC-T cells was also lower in encapsulated isolates while acapsular mutants persisted longer in host cells compared to the wild-type strains [135]. A one herd study reported that acapsular isolates belonging to ST9 exhibit high invasive capacity, a phenotype that was suggested to contribute to the dissemination of bacteria among lactating cows [136].

In summary, the expression of a large array of surface proteins with the ability to bind to extracellular matrix proteins by bovine *S. aureus* highlights the different strategies that have evolved to promote colonization and pathogenesis. Studies have reported the high prevalence of particular adhesins among bovine isolates from intercontinental herds, but further functional analysis is required, along with improved understanding of the synergy between the adhesins, and the regulation of gene expression, to provide important insights into the progression of intramammary infection.

#### Biofilm formation and bovine mastitis

*S. aureus* of bovine origin is usually described as a biofilm producer [109, 137, 138], a trait that is usually related to bacterial persistence and increased tolerance to antibiotics [139]. The biofilm has a complex structure consisting of many cell layers embedded in an extracellular matrix, which in *S. aureus* consists mainly of the polysaccharide intercellular adhesin (PIA), a poly-β (1–6)-N-acetylglucosamine (PNAG) produced by the *ica* locus. Besides PIA, the biofilm matrix is also composed of several MSCRAMMs, such as FnBPs, ClfA, ClfB, and Protein A, that promote bacterial adhesion to host cells to initiate biofilm formation (56). The production of *S. aureus* biofilm has not yet been demonstrated *in vivo* in IMI, despite immunological detection of certain components such as slime and PIA from mammary gland samples [134, 140].

There are many differences in the production of biofilm among bovine isolates of different genetic origins. Generally, strong biofilm producers belong to the *agr*III group [113, 141] and invade epithelial cells to a lesser degree compared to planktonic or low biofilm producers [142, 143]. *agr*I-type isolates are moderate biofilm producers, highly invasive, and are frequently isolated from subclinical mastitis in contrast to the *agr*II group that produces less biofilm, has reduced invasiveness, and is more related to clinical mastitis [144–147]. Nevertheless, differences in the level of biofilm formation among isolates from the same lineage are also observed [23, 24].

Milk and lactose can positively influence biofilm formation by *S. aureus agr*II bovine isolates due to the production of PIA [141, 144, 148]. In addition, milk and lactose stimulate the expression of the lactose transporter EII and the teichoic acid biosynthesis protein B (TagB) that is regulated by Rbf, the repressor of biofilm [148–150]. Teichoic acids are an important component of the staphylococcal biofilm extracellular matrix and establish electrostatic interactions with PIA [151].

*S. aureus* can cause mastitis in cows in different stages of lactation and infections frequently persist over the ongoing lactation [152, 153]. A non-lactating period, also called the dry period, allows the recovery of the cow’s mammary gland and improves milk production in the following lactation. Additionally, dry cow therapy is recommended to reduce the risk of staphylococcal infections. High biofilm producers are likely to persist during the cow’s dry period [23] due to their efficient adherence to the mammary epithelium. Also, *S. aureus* isolated from milk is more likely to produce biofilm compared to bacteria isolated from teat skin and milking unit liners [154]. In a three-year follow-up of a dairy herd, *S. aureus* strains that produced more biofilm and higher amounts of PNAG showed higher within-herd prevalence and persistence [136]. These strains also presented reduced cellular cytotoxicity and high invasiveness. A less prevalent phenotype whose persistence was related to the formation of dendrites instead of biofilm was also described by the authors. These findings show that although biofilm production is usually described as a primordial factor that contributes to persistence other bacterial traits may also support chronic infections during bovine mastitis.

The impact of *ica* deletion on biofilm production can be compensated by the expression of a surface protein known as the biofilm-associated protein (Bap) that promotes adhesion of *S. aureus* to abiotic and biotic surfaces [155]. Isolates expressing Bap adhere to epithelial cells but are less invasive due to the interaction between Bap and the Gp96 receptor expressed by mammary epithelial cells, which interferes with the internalization pathway mediated by FnBP [99, 156]. Furthermore, isolates harboring *ica* and *bap* are strong biofilm producers and are more resistant to antibiotics than isolates harboring only *ica* or *bap* [155]. However, Bap-positive strains can lose the ability to form biofilm when grown in milk, probably due to the stabilization of Bap in the presence of calcium [49]. At lower concentrations of calcium, Bap is cleaved into fragments that form amyloid fibers providing a scaffold for biofilm development [157]. Taken together, these data indicate that *S. aureus* of bovine origin forms biofilm through a PIA-dependent manner during the lactating period but a PIA-independent mechanism via Bap might play a role during the dry cow period when concentrations of calcium in the udder are low.

#### Toxins as important virulence factors in mastitis

A wide array of different secreted toxins has been implicated in *S. aureus* disease pathogenesis [158]. Superantigens (SAgs) are a family of potent immunostimulatory exotoxins produced by *S. aureus* that are known for their ability to circumvent normal immune function. They bind as a bridge to major histocompatibility complex class II molecules and specific Vβ segments of T-cell receptors, resulting in their proliferation, differentiation into effector cells, and massive cytokine release [159]. As a result, several T cells that share the same Vβ segment may be activated, independently of antigen specificity [160]. SAgs in bovine strains of *S. aureus* include TSST-1, staphylococcal enterotoxins (SE), and staphylococcal enterotoxin-like proteins (SEl) that are mostly encoded on MGE [88, 93].

Genomic analysis revealed variation in the gene content of SAgs of bovine isolates from 57 distinct sequence types (ST) [161] in concordance with the findings reported for isolates around the world [90, 162, 163]. However, there are disparities in the distribution of the enterotoxin gene cluster (*egc*), which is highly prevalent within CC30, CC151, and CC45, and the pathogenicity island SaPI_bov_, which is primarily associated with CC133 and CC151 [161]. The prevalence of enterotoxins is also highly variable in isolates belonging to different geographical locations [21, 124, 164], which is explained by the wide variety of *S. aureus* genotypes found in dairy cattle [124], besides differences in environmental and management factors in each geographical area [165].

The presence of specific enterotoxin genes has been linked to acute and clinical bovine mastitis [23, 166] although *seg* and *sei* were found in persistent subtypes recovered from cows presenting subclinical mastitis [136]. Additionally, *seh* and *sek* tend to be more frequent in isolates causing subclinical mastitis, while *sed* and *sej* are mostly associated with persistent mastitis [166]. Other studies reported that neither the genes *sea* and *sed* [167] nor the set of genes *sea, seb, see* [168] were detected in isolates causing subclinical mastitis in cows on dairy farms in Spain [167] or the USA [168]. Due to the large repertoire of enterotoxins present in *S. aureus* more studies are needed before gene signatures can be associated with the outcome of mastitis in dairy cows.

Current evidence indicates the involvement of SAgs in the pathogenesis of bovine mastitis. Enterotoxin M and H contribute to inflammation, necrosis, and/or apoptosis of bovine mammary epithelial cells [169, 170], pathology also observed upon injection of SEC into the mammary glands of mice [171]. However, the high concentrations of toxins used in those studies may not mimic the *in vivo* condition and therefore are unlikely to occur during intramammary infection. It was demonstrated that most SAgs produced by *S. aureus* RF122 present mitogenic activity for bovine T cells, even at low concentrations [161]. In this study cows challenged with a SAg-deficient strain demonstrated an inability to develop clinical mastitis but had similar somatic cell counts and milk quality compared to wild-type RF122-infected cows, highlighting the functional importance of SAgs during bovine infection.

*S. aureus* also produces numerous membrane-damaging toxins that compromise host-cell function *in vitro* and are involved in iron acquisition and host immune evasion [172]. α-hemolysin (Hla) binds to the ADAM10 receptor and assembles into a β-barrel transmembrane pore in the cell surface, promoting the release of small molecules that lead to tissue necrosis [173]. Phagocytized bacteria secrete Hla to lyse bovine endothelial cells, an event that may free intracellular bacteria to target other cells [174]. β-hemolysin (Hlb) also damages the secretory epithelial cells but has less cytotoxicity, with a synergistic effect seen with a combination of both toxins [175]. The hydrolysis of sphingomyelin by β-hemolysin increases host cell permeability with progressive loss of cell surface charge, rendering the cells more susceptible to the action of α-hemolysin. Additionally, β-hemolysin exerts lymphotoxic effects and lyses bovine erythrocytes contributing to iron acquisition [172]. δ-hemolysin (Hld) is a small peptide that is translocated across the plasma membrane at low concentrations, forming a transient structure that perturbs the bilayer and induces ionic efflux [176]. Hld belongs to the family of phenol-soluble modulins (PSM), toxins that are highly cytolytic and have proinflammatory activities. A high expression of *hld* but no other *psm* genes was reported in the well-studied bovine strains RF122 and N305 [177]. In the same study, the authors showed reduced cytokine production in bacteria expressing *psmαβhld*, suggesting that an attenuated immune response is related to chronic mastitis.

The hemolytic activities of Hlb and Hld produced by *S. aureus* isolates recovered from clinical mastitis when grown in the whey of skimmed milk suggest a role for Hld in IMI [178] but there is experimental evidence that supports the relevance of the other toxins in bovine mastitis. Antibodies raised against α- but not β-hemolysin produced by bovine *S. aureus* strains protect rabbits from mastitis [179]. Intramammary injections of Hla in lactating rabbits disrupt the architecture of the mammary gland, with necrotic lesions seen at high doses while injection of purified Hlb causes an influx of polymorphonuclear leukocytes (PMN) into the alveoli besides edema [180]. These findings were further corroborated by studies showing less necrosis and greater preservation of tissue structure 24 h post-challenge in a murine model with a Δ*hla*/Δ*hlb* mutant [181]. Compared to the parental strain that caused 60% mortality, no mortality was seen in mice inoculated with the double mutant. Cows infected with Δ*hla* and Δ*hlb* mutants were able to eliminate the bacteria and suffer only mild inflammation compared to the severe clinical signs presented by animals infected with the parental strains [182]. Taken together, these studies reveal the importance of Hla and Hlb in bacterial virulence during IMI.

Genes involved in the synthesis of hemolysins are frequently found in bovine isolates of *S. aureus* [166, 183, 184] and nearly 50% of those isolated from subclinical disease show hemolytic activity [163, 185]. There is no correlation between hemolysin expression and bacterial lineage since strains from the same ST often demonstrate both high and low levels of gene expression and activity [186, 187]. *S. aureus* isolated from cows with subclinical mastitis that shows higher expression of *hld* are more likely to be nonpersistent during either lactation or through the dry period [23]. Also, isolates causing clinical mastitis show higher hemolytic activity and prevalence of hemolysin genes when compared to isolates recovered from subclinical mastitis [163, 188], which may result in greater damage of the bovine mammary secretory epithelial cells. In addition, the presence of single nucleotide polymorphisms within the promoter region of *hla* is likely to be associated with a high α-hemolysin expression by *S. aureus* isolates associated with severe bovine mastitis, a finding that may be used as a marker to discriminate strain virulence [189].

Leukocidins secreted by *S. aureus* are bicomponent toxins that target white blood cells leading to the formation of pores that disrupt host cell function allowing *S. aureus* to escape from the immune system [190]. The leukocidin S subunit binds to a receptor in the host cell membrane, followed by F subunit recruitment and oligomerization into an octameric structure creating a β-barrel channel inserted in the host cell lipid bilayer. The presence of interchangeable classes of S and F subunits in bovine *S. aureus* raises the hypothesis that leukocidins may present distinct activities according to the cell target during the infective process [191]. LukMF’ is restricted to *S. aureus* strains associated with animals such as bovines and ovines [192] although the prevalence of the *lukM* and *lukF* genes varies considerably (4–96%) among *S. aureus* isolates recovered from cows [82, 193, 194]. Among all bovine-associated *S. aureus* lineages, CC97 showed a high prevalence of the genes *lukMF* [11, 195]. Also, there are reports that associate gene presence with certain clones such as CC151, CC479, and CC133 [36]. Even though the genes have been detected in *S. aureus* isolated from subclinical mastitis [24, 82, 167], expression is more often detected in those recovered from clinical mastitis or lineages that carry a nonsense mutation in the repressor of toxins Rot [82]. The presence of LukMF´ and antibodies in milk and the serum of goats that received intramammary infusion with a high leukocidin-producing strain is evidence of the functional role of these toxins during mastitis [196].

The high cytotoxicity of LukMF’ may be conferred by the high efficiency of LukM for cell receptors present on bovine leukocytes, neutrophils, and macrophages [197]. Interestingly, no inflammation was seen in cows that received intramammary injections of purified LukMF’ [198]. Later, it was shown that the expression of CCR1 on bovine, but not on human neutrophils was the cause for species-specific killing by LukMF’ [199]. Further studies challenged cattle with high and intermediate LukMF’ producing strains and correlated the higher level of expression and the presence of milk antibodies against LukM with clinical mastitis severity [96]. Taken together, these studies strongly support the functional significance of LukMF’ in the pathogenesis of bovine mastitis.

Other leukotoxins such as γ-hemolysin (Hlg) and LukED are produced by bovine *S. aureus* but vary in their activities against bovine cells. These toxins recognize certain receptors in neutrophils but compared to LukMF’ they are expressed in lower levels during *in vitro* growth [96]. Hlg is composed of the F subunit HlgB that combines with HlgC or HlgA to form the active toxin [200]. The effect of HlgAB on bovine PMN is greater than HlgBC [191]. The same authors showed that reduced activity of the leukotoxin LukED was boosted when LukD was replaced by LukM. The genes *hlg* and *lukED* are frequently reported in bovine *S. aureus* [184, 195, 201] but more studies are needed to provide data on their role during IMI.

In summary, bovine strains of *S. aureus* produce a plethora of toxins that target different host cells and have wide ranging roles during infection including nutrient acquisition and immune evasion. If in the past studies were focused on detecting their presence, now they have started to reveal the molecular functions of toxins and their interplay with the host. The immunogenicity of some toxins has been shown supporting their use as therapeutic agents for fighting IMI. Though higher levels of toxin expression are more likely to be associated with more severe symptoms of mastitis it is still unknown if their complementary and/or redundant activities are needed during IMI progression.

## Conclusions and future directions

*S. aureus* has been the subject of intense investigations in the veterinary field and is probably the most studied causative agent of bovine mastitis, a disease with a huge impact on the dairy supply chain. Here we compiled findings specifically related to important virulence factors of bovine *S. aureus*. Considering the 27 countries from which the studies have been published, CC97 and t267 are the most disseminated lineages. There is no clear pattern of distribution of adhesion or toxin genes in bovine *S. aureus* isolates making the outcome of association studies of mastitis and novel therapeutic development complicated. Although the production of biofilm has been reported for every field isolate, more evidence is needed to confirm *in vivo* biofilm formation and the specific role of Bap in bovine *S. aureus* antibiotic-resistant biofilm production. Except for leukocidin LukMF’, few virulence determinants have been correlated with clinical severity.

There is still much must be done to improve animal health, diagnosis, and control of mastitis in dairy herds. 3D cell culture is emerging as a powerful technology to replicate mammary gland structural complexity, allowing advances in the pathophysiology of mastitis and reducing the dependence on animal models. Organoid cultures open new windows to explore the molecular crosstalk between host and pathogen, host and lineage-specific traits that affect disease outcome, and the contribution of the host microenvironment to the expression of the virulence factors highly prevalent in field isolates. This may shed light on the *S. aureus* biology of bovine mastitis and eventually open new perspectives aimed at better controlling bovine mastitis.

While numerous genomes have been sequenced, bovine *S. aureus* contains many genes of unknown function. The construction of transposon (Tn) mutant libraries will deepen our understanding of the pathogen´s biology, unveil new virulence factors and mechanisms of bacterial resistance to antibiotics allowing more efficient strategies for bacterial control. The co-existence of different clonal complexes within dairy herds is a challenge to the control of mastitis. Tn mutagenesis may also be used to identify lineage-specific traits that contribute to the success of clinically relevant clones.

Antibiotics are frequently used to prevent and treat bovine mastitis. With the emergence of antimicrobial-resistant clones and consumer demand for food safety, farmers must rely on strategies other than herd sanitation to control intramammary infections. Although vaccination is available, multifactorial causes like poor antigens and gaps in the understanding of the immune response hinder the development of effective vaccines against *S. aureus*. Over the last years, there has been an increase in the search for alternative treatment methods using natural products, bacteriocins, bacteriophages, and nanoparticles. Despite showing promise, the inhibitory activity seen *in vitro* is not always sustained when the therapy is tested *in vivo* regardless of the animal model used, an interference probably caused by milk components. This can be circumvented using nanocarriers that bring stability, solubility, tissue permeability, or controlled release of the antibiotic drug which can be used in formulations to eradicate planktonic or sessile bacteria. However, there is still a long road to successful clinical translation.

## Data Availability

Not applicable.

## Abbreviations

Bap: Biofilm-associated protein
bMEC: Bovine mammary epithelial cells
CC: Clonal complex
Clf: Clumping factor
FnBP: Fibronectin-binding protein
IMI: Intramammary infection
LukMF’: Bicomponent leukocidin LukMF’
MAC-T: Mammary alveolar cells-large T antigen
MSCRAMMs: Microbial surface component recognizing adhesive matrix molecules
MLST: Multilocus sequence type
MGE: Mobile genetic elements
PIA: Polysaccharide intercellular adhesin
PNAG: Poly-β (1–6)-N-acetylglucosamine
Rbf: Repressor of biofilm
SAgs: Superantigens
TagB: Teichoic acid biosynthesis protein B (TagB)
vWBP: von Willebrand binding protein
